# On solving initial value problems for partial differential equations in maple

**DOI:** 10.1186/s13104-021-05715-4

**Published:** 2021-08-10

**Authors:** Srinivasarao Thota

**Affiliations:** Department of Applied Mathematics, School of Applied Natural Sciences, Adama Science and Technology University, Post Box No. 1888, Adama, Ethiopia

**Keywords:** Initial value problems, Maple implementation, Symbolic algorithm, Partial differential equations, 35F10, 35G10, 15A29

## Abstract

**Objectives:**

In this paper, we present and employ symbolic Maple software algorithm for solving initial value problems (IVPs) of partial differential equations (PDEs). From the literature, the proposed algorithm exhibited a great significant in solving partial differential equation arises in applied sciences and engineering.

**Results:**

The implementation include computing partial differential operator (PartialDiffOperator), Greens function (GreensFunction) and exact solution (ExactSolution) of the given IVP. We also present syntax, ApplyPartialDiffOp, to apply the partial differential operator to verify the solution of the given IVP obtained from ExactSolution. Sample computations are presented to illustrate the maple implementation.

## Introduction

In the scientific area, symbolic computation is one of the significant subjects, and it is playing dominant role in solving many mathematical equations, particularly the applications related to differential equations. In the symbolic computation research, the biggest success is the developing several substantial software systems. Several symbolic algorithms/methods have been created by various scientists, researchers and engineers; see, for example,  [1–26]. Most of these algorithms have been implemented in various mathematical software. Hence, in this paper, we focus on the implementation of the efficient algorithm presented in [13]. For more understanding and application on Maple software commands on how to obtain numerical solutions and the plots see [27–31].

In this paper, we mainly focused on the Maple implementation of the initial value problems (IVPs) for solving partial differential equations (PDEs) with constant coefficients. The proposed algorithm/method was introduced by S. Thota and S. D. Kumar in 2020, see [13] for more details. In [13], they presented a new symbolic method/algorithm to find the Green’s function for a given IVP of linear second order PDEs with constant coefficients. In this method, they focused on computing the Green’s function using the integro-differential algebras [1, 3, 9, 25]. They have discussed several numerical examples to illustrate the symbolic algorithm. They also briefly discussed about the implementation in Maple. The rest of paper is planned as follows. In Sect.  1.1, we briefly state the symbolic algorithm of IVP for PDEs, Sect.  2 presents the Maple implementation of the algorithm with pseudo-code and Maple programming and Sect.  3 focused on sample computations to illustrate the implementation.

### Symbolic method/algorithm

In this section, we recall the symbolic method/algorithm for IVPs for second order PDEs, see [13], for more details about the algorithm.

The general form of the IVP for a second order PDE with inhomogeneous initial conditions over $\mathcal {F} = C^\infty (\mathbb {R}^2)$ is as follows.

1 $$\begin{aligned}&a \frac{\partial ^2 u(x,y)}{\partial x^2} + b \frac{\partial ^2 u(x,y)}{\partial x \partial y} + c \frac{\partial ^2 u(x,y)}{\partial y^2} = f(x,y), \\&u(0,y)=\alpha _1(y),\frac{\partial u(0,y)}{\partial x}=\alpha _2(y), \end{aligned}$$

where $u(x,y) \in \mathcal {F}$ is unknown function which is going to be determined, called solution of (1), using forcing function $f(x,y) \in \mathcal {F}$ and the initial data $\alpha _1(y),\alpha _2(y) \in \mathcal {F}$. The proposed algorithm solves the IVP (1) not only for static values of *f*(*x*, *y*), $\alpha _1(y),\alpha _2(y)$ but give a standard formula of the solution of IVP (1). The operators notation of the IVP (1) is

2 $$\begin{aligned}&\texttt {D}u = f, \\&\tilde{\texttt {E}} u = \alpha _1,\tilde{\texttt {E}} \partial _x u = \alpha _2, \end{aligned}$$

where $\texttt {D}= a\partial _{xx} + b\partial _{xy} + c\partial _{yy}$ is differential operator; $\tilde{\texttt {E}} u = u(0,y), \tilde{\texttt {E}} \partial _x u = \left( \frac{\partial u}{\partial x} \right) _{x=0}$ are initial evaluation operators; and $\alpha _1, \alpha _2$ are initial data of the given IVP. One can factor the second order differential operator into linear operator [13] and then the given IVP for PDE (2) can be expressed as follows

$$\begin{aligned} &a \left( \partial _x + \left( \frac{b}{2a} + \frac{\sqrt{b^2-4ac}}{2a} \right) \partial _y \right) \left( \partial _x + \left( \frac{b}{2a} - \frac{\sqrt{b^2-4ac}}{2a} \right) \partial _y \right) u = f, \\&\tilde{\texttt {E}} u = \alpha _1,\tilde{\texttt {E}} \partial _x u = \alpha _2. \end{aligned}$$

Now we present main theorem of algorithm for computing the Green?s function of IVP (2) over integro-differential algebras.

#### Theorem 1

*Let*$(\mathcal {F},\texttt {D},\texttt {A})$* be an integro-differential algebra. Suppose *$\texttt {D}= a\partial _{xx} + b\partial _{xy} + c\partial _{yy}$* is a linear partial differential operator of second order. Then the IVP* (2)* has the unique solution as follows, for* $m_1 \ne m_2$,

$$\begin{aligned} u =&~ \frac{1}{a}\int _0^x \int _0^t f(\xi , y + m_1(t-x) - m_2(t-\xi )) \ d\xi \ dt \\&~ + \left( \frac{m_2}{m_2 - m_1} \right) \alpha _1(y-m_1x) + \left( \frac{m_1}{m_1-m_2} \right) \alpha _1(y-m_2x) \\&~ + \left( \frac{1}{m_1-m_2} \right) \int _{y-m_1x}^{y-m_2x} \alpha _2(s) \ ds \in \mathcal {F}, \end{aligned}$$

*for* $$m_1 = m_2$$,

$$\begin{aligned} u =&~ \frac{1}{a}\int _0^x \int _0^t f(\xi , y - m_1(x-\xi )) \ d\xi \ dt \\&~ + \alpha _1(y-m_1x) + x \alpha _2(y-m_1x) + m_1 x \partial _y \alpha _1(y-m_1x) \in \mathcal {F}, \end{aligned}$$

*here* $$m_1 = \frac{b}{2a} + \frac{\sqrt{b^2-4ac}}{2a}, m_2 = \frac{b}{2a} - \frac{\sqrt{b^2-4ac}}{2a}$$, *and* $$a \ne 0,b,c \in \mathbb {R}$$.

#### *Proof*

See [13, Theorem 5].$$\square$$

## Main text

This section discusses about the Maple implementation of IVPs for PDEs, IVPforPDE package and its pseudo-code. In this implementation, various data types are created to compute the Green’s function, namely PartialDiffOperator(a1,a2,a3), where a1,a2,a3 are the coefficients of the differential operator as given in Eq. (1); and GreensFunction(ParDiffOp), where ParDiffOp is the partial differential operator as given Eq. (2). Now finally, we have ExactSolution(ParDiffOp, forcefun, alpha, beta), where forcefun is the forcing function, alpha, beta are the initial data. We also created ApplyPartialDiffOp(ParDiffOp,sol) to verify the solution sol. This Maple package is available with example worksheet at http://www.sinivasaraothota.webs.com/research.

### Pseudo-Code

The following pseudo-code gives the exact solution of a given IVP for PDEs of second order.

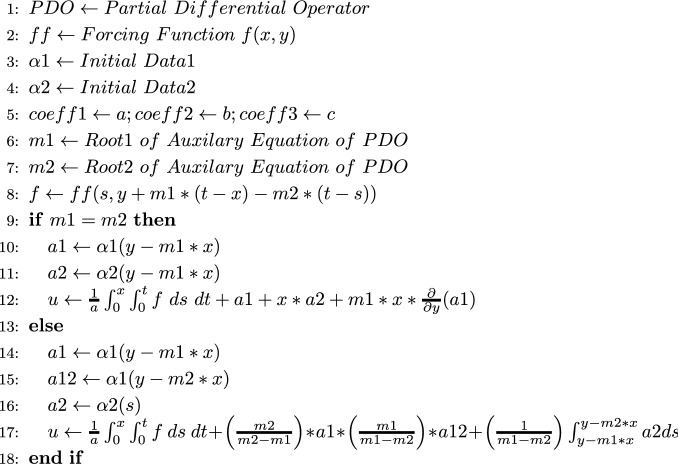



The following pseudo-code shows how to compute the Green?s function of a given IVP for PDEs of second order of the type in Eq.  (1).

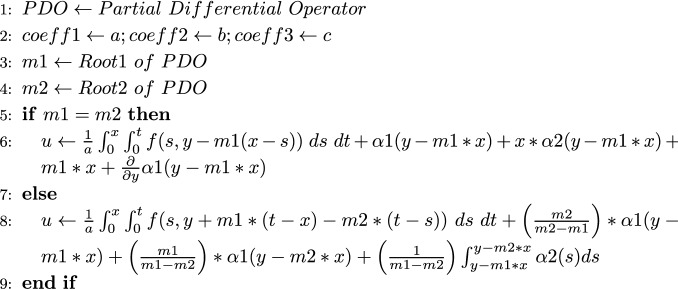



### Maple implementation

The following procedure gives partial differential operator of the given IVP.
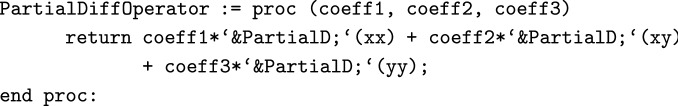


The following procedure produces the Green’s function
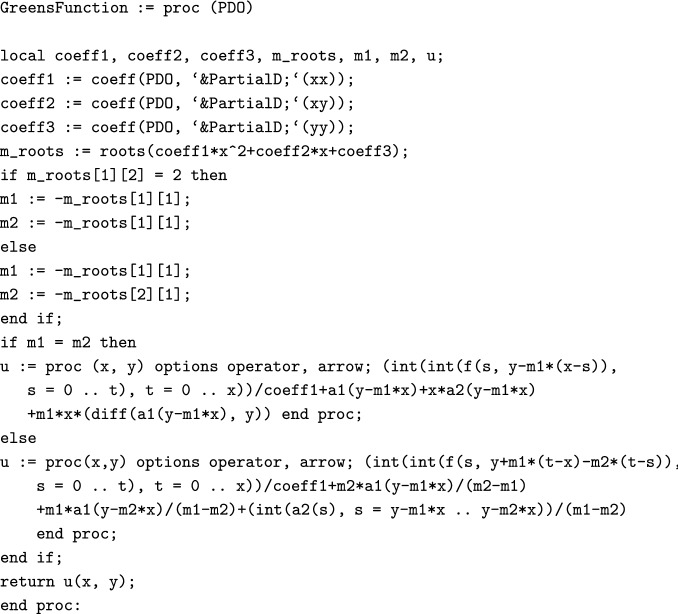


Using the following data type, one can obtain the exact solution of the given IVP.
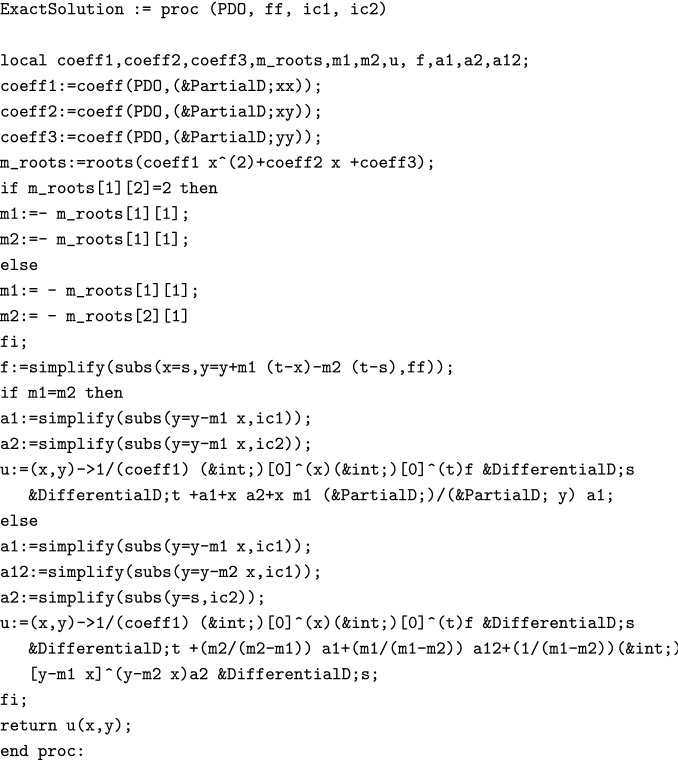


One can also verify the solution using the following procedure.
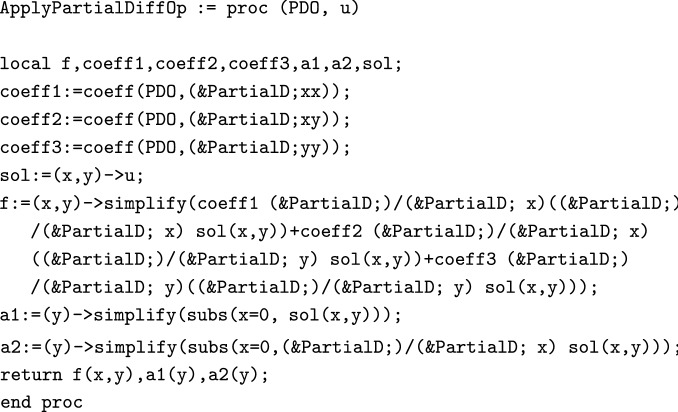


## Results

In this section, we show sample computations using the implementation of IVP. Recall couple of examples from [13] to verify the package. However, there are several example available in [32–34] to verify the Maple implementation.

### Example 1

Consider second order IVP of the form [13] to demonstrate the Maple implementation.

3 $$\begin{aligned} &\frac{\partial ^2 u(x,y)}{\partial x^2} + \frac{\partial ^2 u(x,y)}{\partial x \partial y} - 6 \frac{\partial ^2 u(x,y)}{\partial y^2} = y \cos x, \\&u(0,y) = y-1,\frac{\partial u(0,y)}{\partial x} = y^2. \end{aligned}$$

From Eq. (3), we have $a_1=1, a_2=1, a_3=-6, f(x,y)=y \cos x, \alpha =y-1, \beta = y^2$ Using Maple,

> PDO := PartialDiffOperator(1, 1, -6);

$$\begin{aligned} PDO := \partial (xx)+\partial (xy)-6\partial (yy). \end{aligned}$$

> G := GreensFunction(PDO)

$$\begin{aligned} G =& \int _0^x \int _{0}^{t} f(s, y-2(t-x) +3(s-t)) \ ds \ dt + \frac{3}{5} \alpha _1(y+2x) \\ & + \frac{2}{5} \alpha _1(y-3x) - \frac{1}{5} \int _{y+2x}^{y-3x} \alpha _2(s) \ ds. \end{aligned}$$

> Sol := ExactSolution(PDO, y*cos(x), y-1, y$\wedge$2);

$$\begin{aligned} Sol:= 2y-x-y\cos x+\sin x-1 + \frac{1}{15} (y+2x)^3-\frac{1}{15} (y-3x)^3. \end{aligned}$$

> ApplyPartialDiffOp(PDO, G);

$$\begin{aligned} f(x,y),~a1(y),~a2(y). \end{aligned}$$

> ApplyPartialDiffOp(PDO, Sol);

$$\begin{aligned} y\cos (x),~y-1,~y^2. \end{aligned}$$

### Example 2

Consider second order IVP of the form [13]

4 $$\begin{aligned} &4\frac{\partial ^2 u(x,y)}{\partial x^2} - 4 \frac{\partial ^2 u(x,y)}{\partial x \partial y} + \frac{\partial ^2 u(x,y)}{\partial y^2} = 16 \log (x+2y), \\ & u(0,y)=0, \ \frac{\partial u(0,y)}{\partial x}=0. \end{aligned}$$

The exact solution of IVP (4) as

$$\begin{aligned} u(x,y) =&~ 2x^2 \log (x + 2y). \end{aligned}$$

Using the Maple implementation, we have:

> PDO:=ParialDiffOperator(4,-4,1);

$$\begin{aligned} PDO := 4 \partial (xx) - 4 \partial (xy) + \partial (yy). \end{aligned}$$

> g:=GreensFunction(PDO);

$$\begin{aligned} g:=&~ \frac{1}{4} \int _{0}^{x} \int _{0}^{t} f(s, y+\frac{1}{2}x - \frac{1}{2} s) \ ds \ dt \\&~ + a1(y+\frac{1}{2} x)+xa2(y+\frac{1}{2} x) - \frac{1}{2} x D(a1) (y+\frac{1}{2} x). \end{aligned}$$

> u:=ExactSolution(PDO,16*log(x+2*y),0,0);

$$\begin{aligned} u:=&~ 2 ln(x+2y) x^2. \end{aligned}$$

> ApplyPartialDiffOp(PDO,u);

$$\begin{aligned} 16 ln(x+2y),0,0. \end{aligned}$$

> ApplyPartialDiffOp(PDOp,g);

$$\begin{aligned} f(x,y),a1(y),a2(y). \end{aligned}$$

## Limitations

The algorithm in [13] is focused on the IVP for regular linear PDEs, hence the implemented maple package, IVPforPDE, presented in this paper is valid for the regular linear PDEs with initial conditions. We have also presented a syntax to check the validity of solution of a given problem.

## Data Availability

The datasets generated and analyzed during the current study are presented in this manuscript.

## Abbreviations

IVP: Initial value problem
PDE: Partial differential equation
